# Effector-Mediated Suppression of Programmed Cell Death by *Phytophthora palmivora* in Oil Palm

**DOI:** 10.3390/jof10110750

**Published:** 2024-10-30

**Authors:** María Camila Rodríguez-Cruz, Carmenza Montoya, Iván Ayala-Diaz, Leonardo Araque, Hernán Mauricio Romero

**Affiliations:** 1 Biology and Breeding Research Program, Colombian Oil Palm Research Center, Cenipalma, Calle 98 No. 70-91, Piso 14, Bogotá 111121, Colombia; mcrodriguez1508@gmail.com (M.C.R.-C.); cmontoya@cenipalma.org (C.M.); iayala@cenipalma.org (I.A.-D.); laraque@cenipalma.org (L.A.); 2 Department of Biology, Universidad Nacional de Colombia, Bogotá 111321, Colombia

**Keywords:** effector proteins, programmed cell death (PCD), plant–pathogen interactions, bud rot, transient expression assays, *Phytophthora palmivora*, oil palm

## Abstract

*Phytophthora palmivora* is the pathogen causing bud rot in oil palm *(Elaeis guineensis*). This pathogen secretes effector proteins that manipulate host defenses, contributing to disease progression. In this study, we systematically investigated the role of specific effector proteins in suppressing programmed cell death (PCD) in oil palm leaflets. Our approach included using genomic and transcriptomic data from a Colombian *P. palmivora* isolate alongside the coexpression network of a substantial effector dataset. From this analysis, ten candidate effectors were selected, characterized, and evaluated for their ability to suppress PCD in oil palm leaflets through transient expression via biolistics. Several effectors exhibited significant anti-PCD activity in susceptible and less susceptible oil palm genotypes. Notably, the effectors Avr3F (689), RxLR (1540), and RxLR (1546) demonstrated suppression of PCD in both genotypes, while the other effectors played variable roles in PCD regulation. Phylogenetic analysis further identified distinct clades among the effectors, possibly associated with their functional activities. Additionally, specific motifs, such as RXLR-dEER, K, and Y, appeared to correlate with PCD suppression. This research enhances our understanding of the molecular mechanisms underlying the interaction between *P. palmivora* effectors and oil palm host responses, highlighting these proteins’ genotype-specific regulation of PCD. The findings contribute valuable insights into plant–pathogen interactions and offer potential avenues for targeted disease control strategies in the oil palm industry.

## 1. Introduction

The African oil palm (*Elaeis guineensis* Jacq) is one of the most productive oil crops, requiring less planted area, fewer pesticides, and less fertilizer than other crops do to produce the same amount of oil [1]. As of 2022, global oil palm production reached 79.16 million metric tons (Mn T), and the leading producers were Indonesia, Malaysia, Thailand, and Colombia, which ranked fourth with a production of 1.77 million metric tons per year [2]. However, significant limiting factors affect oil palm productivity, particularly diseases. In Colombia, one of the primary limiting factors is a disease known as bud rot, which is caused by the oomycete *Phytophthora palmivora* (E.J. Butler) [3]. *P. palmivora* is considered one of the most significant plant pathogens due to its wide range of hosts, which includes economically important species. *P. palmivora* has been identified as the causal agent of various diseases, including pod rot of cocoa, root rot of papaya, and bud and stem rot of oil palm [4,5,6]. Oil palm bud rot disease has had a significant economic impact, resulting in approximately USD 250 million in losses in the central region [3]. Despite the ongoing efforts to develop strategies against bud rot, these measures are generally not definitive solutions to curb pathogen proliferation.

A perdurable solution for bud rot involves the utilization of resistant cultivars. However, there is no conclusive evidence of resistance sources in African oil palm [7]. Resolving the health and sanitary issues related to oil palm cultivation is a priority, and this can be achieved through the study and resistance characterization of bud rot in wild germplasm, as well as the incorporation of resistance genes into commercial materials. Nevertheless, due to oil palm’s perennial nature, traditional breeding methods require significant time and financial investments [8,9]. An alternative approach to expedite the discovery of resistance genes involves a comprehensive understanding of the pathogen genome, particularly the characterization of proteins known as effectors. The pathogen employs these effectors to evade plant defenses and manipulate the host metabolism. This characterization process, known as effectoromics, uses pathogen effectors [10] to assess various plant germplasms and identify disease resistance sources [11].

The African oil palm genome comprises 34,802 genes [12], whereas the *P. palmivora* genome comprises 45,416 genes [13,14]. Among these pathogen genes, specific genes, including virulence proteins known as effectors, are responsible for the pathogenicity process [15]. Effectors are proteins that facilitate the colonization process of plants and aid in evading recognition. When a plant detects a foreign element, a defense response is triggered. The effectors play a role in subverting this recognition process [16]. These defense responses include various reactions, such as the production of reactive oxygen species (ROS), the deposition of callose, the transcription of defense-associated genes, and the induction of a hypersensitive response (HR), leading to cell death [17].

Oomycete effectors can be categorized into two main groups: apoplastic effectors and cytoplasmic effectors. Apoplastic effectors are characterized by their accumulation in the plant’s intercellular spaces, known as the apoplast. On the other hand, cytoplasmic effectors are directly translocated into the plant cell through a specialized infection structure called a haustorium [18]. Apoplastic effectors consist of secreted hydrolytic enzymes such as proteases, lipases that probably degrade plant tissue, and enzyme inhibitors to protect against plant defense enzymes and necrotizing toxins [19].

Among the cytoplasmic effectors, two prominent groups are CRNs and RXLRs. CRN (crinkling necrosis) effectors have been identified in all sequenced plant-pathogenic oomycetes [20,21]. They possess a characteristic N-terminal region with an LXLFLAK motif, followed by a conserved DWL domain. These effectors are associated with a hemibiotrophic lifestyle [22] and tend to accumulate in the nucleus, leading to necrosis [23]. Moreover, RXLR effectors contain an amino-terminal motif represented by Arg-X-Leu-Arg, where X represents any amino acid [21,22,24]. This motif is typically located in the N-terminus within the first 40 amino acids of the protein, following a signal peptide. It is often followed by an EER (Glu-Glu-Arg) motif [25]. Many RxLR effectors report conserved C-terminal motifs, called W, Y, or L, which usually play a role in suppressing host defenses or recognition by R proteins [26]. RXLR effectors are the most abundant group, with a repertoire ranging from 350–563, depending on the species [22].

In the case of *P. palmivora*, several studies have reported on the repertoire of effectors. Gil et al. [13] identified 1,400 candidate effectors in the *P. palmivora* genome. Among these, 799 were classified as RxLR effectors, and eight were classified as CRN effectors after their signal peptide sequences were analyzed and classified as secreted proteins. Similarly, Ali et al. [14] identified 414 RxLR and four CRN effectors with predicted signal peptide sequences. In another study, Evangelisti et al. [27] reported 140 RxLR and 15 CRN effectors based on a de novo transcriptome assembly of a Colombian *P. palmivora* isolate, focusing solely on the predicted secretome.

Genetic transformation is a commonly employed technique for functional characterization of the effector repertoire of different host plant materials. Genetic transformation involves incorporating external genetic material (genes) into the target organism’s genome, enabling the evaluation of the organism’s response to the presence of these genes. Various transformation methods have been reported, which utilize two main strategies. The first strategy involves using the bacterium *Agrobacterium tumefaciens* as a vector to introduce the desired genetic material into the organism’s genome [28,29]. The second strategy involves biolistic particle bombardment for gene insertion [30,31].

In oil palm, transient transformation studies of target genes have been conducted in various tissues [32] via the biolistic gene gun method [33,34,35]. A co-bombardment approach is employed to evaluate effectors through biolistic bombardment, where a reporter gene and the target gene are simultaneously introduced via a double-barreled particle bombardment device [31]. The commonly used reporter gene in these studies is the β-glucuronidase (GUS) gene. GUS produces a blue precipitate in the transformed cell region when expressed in living cells. However, when genes that induce the plant hypersensitive response (HR) are present, fewer viable cells express GUS, resulting in reduced blue precipitate formation compared with that in the control. The proapoptotic protein BAX is used as a control for programmed cell death (PCD) [36]. BAX was initially isolated from mice and has been used in plants such as tobacco [37] and *Arabidopsis thaliana* [36] to induce a cell death response. BAX has been widely employed for PDC control in various studies, particularly those investigating effector functions [38].

Knowledge of the cytoplasmic repertoire of *P. palmivora* effectors and their study in planta would significantly advance oil palm breeding and bud rot control. This knowledge can also be applied to other commercially important crops affected by this significant plant-pathogenic organism. Therefore, the primary objective of this study was to establish a comprehensive catalog of *P. palmivora* effectors involved in oil palm infection and to assess the transient expression methodology of cytoplasmic effectors in the leaflets of different oil palm genotypes. The above information can be used to select diverse wild germplasms through an effectoromic approach.

## 2. Materials and Methods

### 2.1. Effector Selection

The genome sequencing data [13] and *P. palmivora*–oil palm transcriptome data [39] belong to the Colombian *P. palmivora* isolate, PpalZC01, collected from oil palm-affected tissues and obtained from the CENIPALMA microorganism collection. A list of 310 genes differentially expressed in the PpalZC01 *P. palmivora* isolate from oil palm clones with contrasting responses, specifically Ortet 34 (resistant) and Ortet 57 (susceptible), was generated [40]. Among these genes, 17 were predicted to be RXLR effectors via the PFAM and OmicsBox databases. The list of differentially expressed RXLR effectors was further examined to confirm their classification as cytoplasmic effectors. This confirmation was achieved via EffectorP 3.0 [41] and apoplast predictors [42] to determine which genes corresponded to cytoplasmic or apoplastic-secreted effectors. The Secret Santa package of the R programming language was also employed [43] to establish which cytoplasmic effectors were secreted outside the cell. SignalP v3.0 (http://www.cbs.dtu.dk/services/SignalP, accessed on 28 November 2019) was also used to predict secretion signal peptides for each effector.

Furthermore, the hub score of the coexpression network derived from *P. palmivora* data was used to select a cytoplasmic effector [44]. Consequently, a catalog of 10 cytoplasmic effectors with specific features was selected for further characterization through transient biolistic-mediated expression (Table S1).

Each primer was designed once the cytoplasmic effectors were selected (Table S1). The entire N-terminal region to the signal peptide cleavage region was manually reduced. The Primer3 program was subsequently used to design the primers. ATG and a stop codon were manually added to forward and reverse primers. All primer pairs were tested via the OligoAnalyzer tool from IDTtools (https://www.idtdna.com/calc/analyzer, accessed on 16 February 2020). Primer specificity was tested with primerBLAST, and directionality was tested with Sequencher 5.2.4. The sequences attB1 (GGGGACAAGTTTGTACAAAAAAGCAGGCTTC) and attB2 (GGGGACCACTTTGTCAAAGAAAGCTGGGTC) were added to each primer sequence to the forward and reverse primers, respectively, because the effectors were cloned and inserted into Gateway vectors (Invitrogen technology independent of restriction enzymes).

### 2.2. Alignment and Structure Prediction

Multiple alignments of 10 effector sequences were generated via the ClustalW program [45], with minor manual adjustments to optimize the alignment. The phylogenetic analysis was conducted via MEGA X [46]. A phylogenetic tree was built utilizing full-length amino acid sequences via neighbor-joining [47]. Bootstrap values were calculated based on 1000 replications. The EU282487 Avh331 protein of *Phytophthora sojae* was used as an outgroup for phylogenetic analysis. The sequences were manually inspected to confirm the presence of a distinct RXLR-dEER domain; K, W, and L motifs; and an N-terminal signal peptide.

### 2.3. Plasmid Construction

Cytoplasmic effector (RxLR) genes without a signal peptide were PCR-amplified from PpalZC01 *P. palmivora* isolate DNA via the primers listed in Table S1. All the amplified fragments were purified with a 30% PEG 8000/30 mM MgCl2 solution of Invitrogen Gateway PB clonase II enzyme mixture (Invitrogen™, Waltham, MA, USA) and cloned and inserted into the pDONR207 vector donated by Dr. Camilo López (Manihot Biotec, Universidad Nacional de Colombia, Bogotá, Colombia) via BP recombination (Invitrogen™). Effector constructs and an entry vector (pDONR207) were then used for LR recombination (Invitrogen™) into the expression vector pANIC5B designed for microparticle bombardment. The plasmid used for the transient expression experiments consisted of pANIC5B, a construct harboring the GUSPlus visual reporter cassette, which codes for ß-glucuronidase (GUS) under the control of the constitutive switchgrass polyubiquitin 1 promoter; the overexpression cassette of the target sequence attR1-Cmr-ccdB-attR (Arabidopsis Information Resource (TAIR), Ohio State University, Columbus, Ohio, USA); and the plant selection cassette hygromycin B phosphotransferase coding region (hph). The PDC control protein, BAX protein, in the binary vector pich86988::BAX, which was donated by Zhensheng Kang and Tang Chunlei (Laboratory of Crop Stress Biology for Arid Areas and College of Plant Protection, Northwest A&F University, Yangling, China), was inserted into the expression vector pANIC5B. Individual colonies of 10 effector constructs (S1) were tested via PCR and verified via ABI sequencing at Macrogen INC (Seoul, South Korea).

### 2.4. Plant Material

For the transient expression assay, immature leaflets of adult palm trees were used. Oil palm cultivars with contrasting susceptibilities to bud rot were selected: cultivar *E. guineensis* DXD Code 48, which is known to be susceptible in the field, and cultivar *E. guineensis* DXD Code 82, which is considered less susceptible (possibly resistant) in the field. The immature leaflets were obtained from palm bud cylinders at the Centro Experimental Palmar de la Vizcaína (CEPV, Santander, Colombia). These leaflets were then disinfected with 70% ethanol. The immature leaflets were subsequently separated and selected. Leaflets seven centimeters in length were cut from the distal part and stored in humid chambers, which were Petri dishes with moistened sterile towels. The leaflets remained in the humid chambers until bombardment.

### 2.5. Particle Co-Bombardment of Oil Palm Leaflet Assays

The particle co-bombardment assays were performed on immature oil palm leaflet segments (3.5 cm × 7.0 cm) via a Bio-Rad He/1000 particle delivery system (Hercules, CA, USA) with a double-barreled extension attached, similar to the method described by [48], which enables simultaneous bombardment of two DNA preparations onto the leaflets. Plasmid DNA was precipitated onto gold particles and bombarded according to the protocols provided for the Biolistic PDS-1000/He particle delivery system (Bio-Rad, Hercules, CA, USA) and [49] with minor modifications. Previous standardization procedures were conducted. Briefly, 50 µL of a gold particle mixture diluted to 60 µg/µL (prepared in 50% ethanol) was vigorously vortexed. Then, 10 µL of DNA (5 µg), 50 µL of 2.5 M CaCl2, and 20 µL of 0.1 M spermidine were added, and the mixture was vortexed for 2 min. The MCs were allowed to settle for 20 min at 4 °C and then pelleted by spinning for 3 s in a microfuge. After removing the liquid, the pellet was washed twice with 250 µL of 100% ethanol. Subsequently, 60 µL of 100% ethanol was added, and the pellet was resuspended by vortexing. Finally, 6 µL of the 0.5 µg DNA-coated microcarrier suspension was loaded into the center of a macrocarrier, air-dried, and used for bombardment.

Bombardment was performed via the Bio-Rad He/1000 particle delivery system. The distance from the stopping screen to the target shelf was 60 mm. The distance between the rupture disk and the macrocarrier was adjusted to 1/4 inch, and the highest position in the macrocarrier assembly was used for the microcarrier. Rupture disks with a pressure rating of 1100 p.s.i. were used. The chamber vacuum was maintained at 26 In Hg. The double-barreled extension was cleaned with 70% ethanol and dried between shots.

The bombarded oil palm leaflets were incubated in darkness at 25 °C for 2 days. The samples were subsequently stained for 14–18 h at 37 °C and 100–200 rpm with a solution containing 0.5 mg/mL X-gluc (5-bromo-4-chloro-3-indolyl-β-D-glucuronic acid, cyclohexylammonium salt), 50 mM sodium phosphate (pH 7.0), 0.5 mM K4 Fe(CN)6, 0.5 mM K3Fe(CN)6, 10 mM Na2EDTA (pH 8.0), 14% (*v*/*v*) methanol, and 0.1% (*v*/*v*) Triton X-100. The stained, bombarded leaflets were scanned to count the blue spots representing transformed events. These events were quantified in mm^2^ by scanning the leaflets via an EPSON Expression 1000XL scanner with a resolution of 1200 ppi. The counting of blue points was performed through an image processing pipeline developed in Python 3.9, which identifies point features by segmenting areas within a color range HSV scale, clustering these colors in areas via K-means, and defining edges using the waterseed methodology and mathematical morphology. The segmented areas are then conformed in their color dispersion blob-like pattern by a Laplacian of Gaussian (LoG) kernel, thereby obtaining the point count.

Combination assays were performed to evaluate the functional characterization of the repertoire of ten cytoplasmic effectors in different oil palm cultivars to determine whether an effector could suppress cell death triggered by BAX. Two assays, namely indirect and direct assays, were conducted for this purpose. In the direct assay, one barrel contained a suppressor preparation, Effector + GUS + BAX + GUS, while the other barrel contained a cell death preparation, BAX + GUS + EV + GUS (empty vector). Two assay results were compared using the indirect assay to determine the relative impact. The first assay evaluated the suppressor preparation: Effector + GUS vs. control preparation: EV + GUS, and the second assay evaluated the cell death preparation: BAX + GUS + EV + GUS vs. control preparation EV + GUS. Fifteen pairs of shots were performed for each effector comparison. To assess the statistical significance of the blue spots, the results were evaluated via the Wilcoxon rank sum test for the indirect assay and the Wilcoxon signed-rank test for the direct assay. These experimental procedures enabled the evaluation of the functional characterization of the cytoplasmic effectors in different oil palm cultivars. The statistical tests provided a quantitative analysis to determine the significance of the observed differences in blue spot formation, indicating the efficacy of the effectors in suppressing cell death.

## 3. Results

### 3.1. Effector-Mediated Suppression of PCD Induced in Oil Palm Leaflets

**Figure 1:**
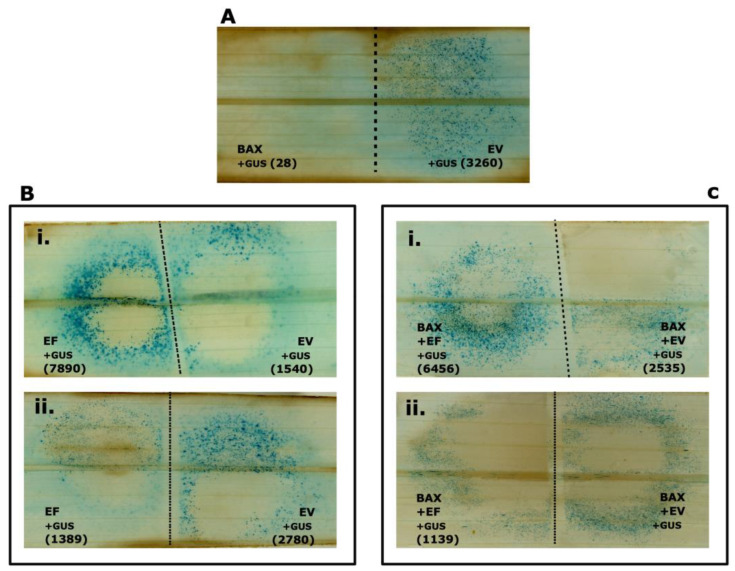
Measuring the suppression of BAX-mediated PCD by *P. palmivora* effectors via double-barreled particle bombardment of 3.5 cm × 7.0 cm immature oil palm leaflet segments. (**A**–**C**) Leaflets bombarded with the pairs of DNA mixtures indicated. BAX: control for PCD; EV: Empty vector; EF: effector tested; GUS: visual reporter. The dotted line indicates the divider position used to prevent overlap of the two bombardment areas. Blue spots indicate GUS expression, which is inhibited by PCD. The number of counted blue spots is shown in parentheses. The conclusions are based on a statistical analysis of results from 15 leaflets similar to those illustrated (Table 1). (**A**) Ablation of blue spots by BAX-triggered PCD, reducing the number of blue spots. (**B**) A control experiment verifying that the effector can (**i**) and cannot (**ii**) significantly increase the number of blue spots in the absence of BAX. (**C**) Suppression (**i**) and induction (**ii**) of BAX-triggered PCD by effectors.

To determine whether *P. palmivora* effectors contribute to virulence in oil palm, they were tested to explore whether they could suppress PCD induced by the proapoptotic protein BAX. The double-barreled attachment of the Bio-Rad Gene Gun was used for the co-bombardment assay in oil palm. We introduced DNA encoding the ß-glucuronidase (GUS) reporter gene into the pANIC5B-GUS plasmid and then into oil palm leaflet cells. When numerous blue spots were observed on oil palm leaflets, they represented the number of living cells expressing GUS (Figure 1A). In contrast, when transient expression was performed with DNA encoding an elicitor of PCD and GUS, PCD decreased the number of living cells expressing GUS (Figure 1A) [30]. When a third gene is introduced on the same particle, two situations can occur: First, the gene can have the ability to suppress PCD, and the successful expression of this gene leads to the recovery of GUS expression in cells, resulting in a more significant number of blue spots. In other words, the introduced gene can suppress the plant defense system (Figure 1(Bi,Ci)). Second, the gene can trigger PCD, and the expression of the gene reduced the number of GUS-positive blue spots, confirming that the gene can trigger PCD in oil palm cells (Figure 1(Bii,Cii)).

The effectors were subsequently co-bombarded with BAX to determine whether they could enhance or suppress the PCD triggered by BAX in oil palm leaflets. In the first barrel, pANIC5B-effector, pANIC5B-BAX, and pANIC5B-GUS were combined, whereas in the second barrel, we combined the pANIC5B-Empty vector, pANIC5B-BAX, and pANIC5-GUS as controls. The number of blue spots for each bombardment was counted, and a Wilcoxon signed-rank test was used to analyze the significant differences between different pairs of samples. Oil palm leaflets co-bombarded with pANIC5B-Effector, pANIC5B-BAX, or pANIC5B-GUS presented significantly (*p* < 0.1) fewer blue spots than did the control, from which we conclude that the effector enhances the PCD triggered by the mouse BAX gene in oil palm.

**Figure 2:**
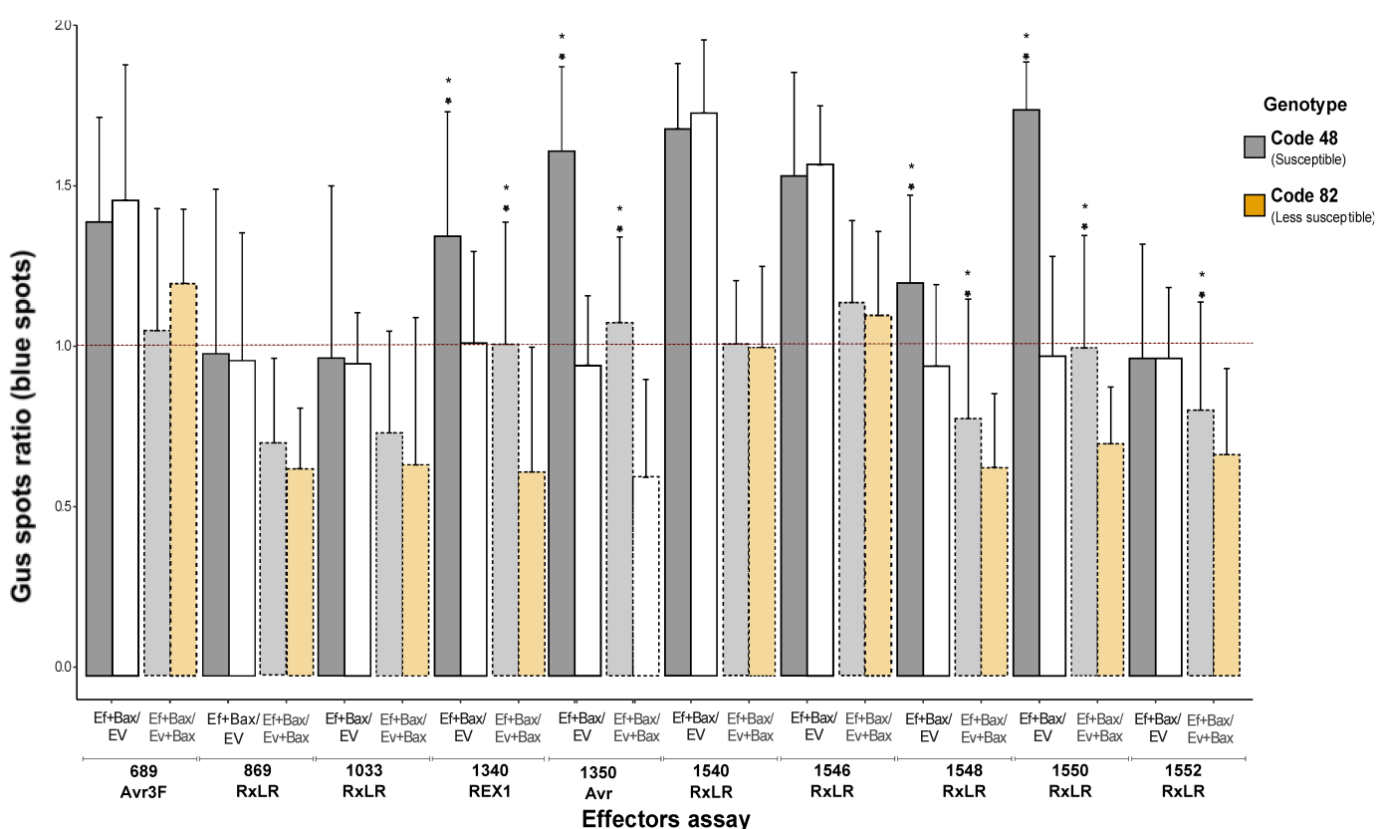
Suppression of BAX-mediated PCD by diverse predicted effectors in *E. guineensis* oil palm leaflets. The quantification of suppression was performed by indirect_(EF+BAX/EV)_ and direct_(EF+BAX/EV+BAX)_ co-bombardment assays. For the indirect assay (dark color bars), the number of GUS-expressing spots surviving in the presence of the 10 effectors (689-Avr3F, 896-RxLR, 1033-RxLR, 1340-REX1, 1350-Avr, 1540-RxLR, 1546-RxLR, 1548-RxLR, 1550-RxLR, and 1552-RxLR) on two genotypes, oil palm susceptible (code 48) (dark gray bars) and less susceptible (Code 82) (dark orange bars), are shown. The results for the direct assay are shown as light gray bars (Code 48) and light orange bars (Code 82). The outcomes that were significantly different according to the Wilcoxon signed-rank test (direct assays) or the Wilcoxon rank sum test (indirect assays) are shown in Table 1. (*) Denote statistical significance differences between genotypes with *p* < 0.05.

Figure 2 and Figure S1 and Table 1 show the results of indirect and direct assays used to assess the anti-PCD activity of different effectors in oil palm genotypes (susceptible 48 and less susceptible 82). The values represent the GUS (β-glucuronidase) expression ratio under various experimental conditions, indicating cell survival and PCD suppression levels. In the indirect assay, the expression levels of GUS in the presence of effectors were compared to those in the control condition (EV + GUS). A ratio greater than 1 indicates potential anti-PCD activity, as the effectors might have suppressed BAX-mediated PCD. The direct assay directly evaluated the ability of the effectors to suppress BAX-mediated PCD. The ratio of (BAX + Effector + EV)/(BAX + EV) was calculated to determine whether the presence of the effectors could restore GUS-expressing cells that were affected by BAX-induced PCD.

Among the tested effectors, the effectors Avr3F (689), REX1 (1340), Avr (1350), RxLR (1540), RxLR (1546), and RxLR (1550) had significant anti-PCD activity in both indirect and direct assays, with ratios greater than 1 (Table 1). These findings indicate that these effectors effectively suppressed BAX-induced PCD and restored GUS-expressing cells. Nonetheless, the effector RxLR (1548) showed moderate anti-PCD activity in the indirect assay but influenced PCD in the direct assay; neither was significant. These findings suggest that these effectors may have indirect roles in PCD regulation. However, the effectors RxLR (869), RxLR (1033), and RxLR (1552) did not show anti-PCD activity in either the indirect or direct essays, with ratios less than 1 (Table 1). These findings suggest that these effectors play a direct role in PCD regulation.

The less susceptible oil palm genotype interacting with the effectors Avr3F (689), RxLR (1540), and RxLR (1546) exhibited significant anti-PCD activity both indirectly and directly. However, the effector REX1 (1340) had a moderate anti-PCD effect in the indirect assay but did not significantly impact the direct assay. Similarly, the effectors RxLR (896), RxLR (1033), RxLR (1350), RxLR (1548), RxLR (1550), and RxLR (1552) did not have a significant anti-PCD effect on either the indirect or direct essays with ratios less than 1 (Table 1). These results demonstrate that certain effectors can potentially suppress BAX-induced PCD in specific oil palm genotypes, whereas others may play more nuanced or indirect roles in PCD regulation.

### 3.2. Effect of Effectors on PCD Induced in Oil Palm Leaflets: A Comparative Analysis of Genotype Responses

A comparative analysis was conducted between the two genotypes to evaluate the potential differences in their response to effectors. The effective compounds Avr3F (689), RxLR (1540), and RxLR (1546) showed significant anti-PCD activity in both the susceptible (48) and the less susceptible (82) genotypes in the indirect and direct assays. These findings indicate that these effectors effectively suppressed BAX-induced PCD and restored GUS-expressing cells in both genotypes. In contrast, the effectors RxLR (896), RxLR (1033), and RxLR (1552) did not have a significant anti-PCD effect on either the indirect or direct essays for both the susceptible (48) and the less susceptible (82) genotypes. These findings indicate that effectors might not be involved in suppressing BAX-induced PCD in these genotypes.

The effector RxLR (1548) showed moderate anti-PCD activity in the indirect assay for genotype 48 (susceptible). However, it did not have a significant effect in the direct assay. Similarly, for the less susceptible genotype (82), the effector RxLR (1548) did not have a significant anti-PCD impact in indirect or direct assays. These findings suggest that the effector RxLR (1548) may have a more indirect or variable role in PCD regulation across genotypes. In contrast, the effector REX1 (1340) showed anti-PCD activity in the direct assay for genotype 48. However, PCD had a significant indirect and direct effect on genotype 82 (less susceptible), suggesting a variable role in PCD regulation across genotypes. Finally, the effectors Avr (1350) and RxLR (1550) displayed significant anti-PCD activity in both the indirect and direct assays for genotype 48. However, in genotype 82, these effectors did not have an anti-PCD effect in the direct assay, although they had significant anti-PCD activity in the indirect assay. These findings indicate that the effectors Avr (1350) and RxLR (1550) may play more direct roles in PCD regulation in the susceptible genotype (48) than in the less susceptible genotype (82).

### 3.3. Suppression of PCD by Diverse Predicted Effector Containing Motifs

**Figure 3:**
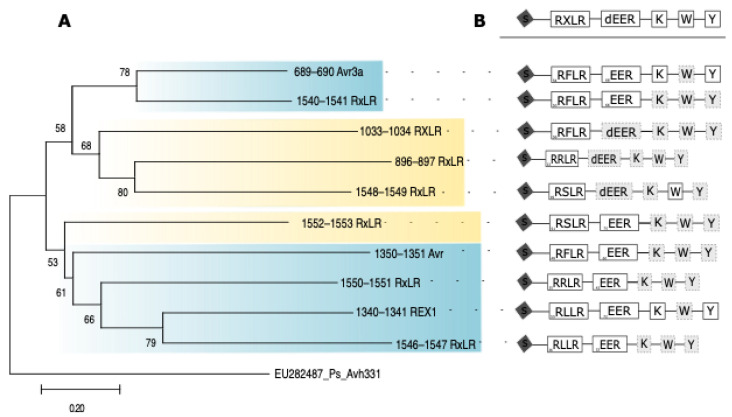
Phylogenetic analysis of the deduced amino acid sequences of effector proteins and diverse predicted effectors containing motifs. (**A**) A representative phylogenetic tree of 10 effectors constructed via full-length amino acid sequences (accession numbers PQ478062-PQ478072) and the neighbor-joining method. Bootstrap values (>100%) based on 1000 replications are shown at branch nodes. EU282487_Ps Avh331 was used as an outgroup. (**B**) The structure of the predicted effector proteins. The positions of each motif are approximately scaled. S, secretory leader; RxLR, dEER motif; K, K motif; W, W motif; L, L motif (Jiang et al., 2008). Dotted outlines and gray lettering indicate weak matches to the respective motifs.

Phylogenetic analysis of the full-length amino acid sequences revealed distinct clades among the effectors. In the first major clade, a minor subclade comprising Avr3F (689) and RxLR (1540) presented the closest relationship, both of which were associated with anti-PCD activity in both susceptible (48) and less susceptible (82) oil palm genotypes (Figure 3A). Within this major clade, a separate subclade containing RxLR (1033), RxLR (896), and RxLR (1548) showed proximity. Notably, these effectors lacked anti-PCD activity in both oil palm materials. A distinct cluster emerged in the second major clade, consisting of the inner effectors Rex1 (1340) and RxLR (1546), which displayed a closer relationship. These effectors predominantly exhibited anti-PCD activity in both susceptible (48) and less susceptible (82) materials. Subsequently, RxLR (1550) and Avr (1350) branched off externally. These effectors demonstrated anti-PCD activity in susceptible (48) material but not in less susceptible (82). Finally, the effector RxLR (1552) occupied an outer branch, indicating a lower level of relatedness within this clade. This effector did not exhibit anti-PCD activity in either material (Figure 3A).

Figure 3B provides an overview of the predicted effector proteins, highlighting the presence of specific motifs within these effectors. Among the effector proteins examined, such as Avr3a (689) and REX1 (1340), the presence of the RXLR-dEER, K, and Y motifs is evident. On the other hand, effectors including RxLR (1540), RxLR (1552), Avr (1350), RxLR (1550), and RxLR (1546) exhibit RXLR-dEER motifs without additional K and Y motifs. In contrast, the effectors RxLR (1033), RxLR (896), and RxLR (1548) are characterized primarily by the presence of the RxLR motif.

## 4. Discussion

The functional characterization of cytoplasmic effectors is essential for understanding the molecular mechanisms that drive *Phytophthora palmivora*’s pathogenesis and its adaptation to hosts. Within *P. palmivora*, a gene set encodes approximately 799 potential RxLR effectors, as reported by Gil et al. [13], raising questions about their shared roles in virulence enhancement. The conservation of effector genes offers valuable insights into virulence mechanisms and could aid in developing long-term resistance strategies by identifying resistance genes that target these conserved effectors [26].

In this study, we investigated the functions of ten cytoplasmic effectors (689-Avr3F, 896-RxLR, 1033-RxLR, 1340-REX1, 1350-Avr, 1540-RxLR, 1546-RxLR, 1548-RxLR, 1550-RxLR, and 1552-RxLR) across different oil palm genotypes. We evaluated their ability to suppress cell death induced by the BAX protein, which is known to trigger programmed cell death (PCD) in plants. BAX is closely associated with the defense-related hypersensitive response (HR) and has been a valuable tool for screening pathogen effectors that inhibit defense-associated PCD [50,51].

Our double-barrel particle bombardment assays revealed varying anti-PCD activity among the effectors in different oil palm genotypes, highlighting the complexity of interactions between pathogen effectors and host defense mechanisms [52].

### 4.1. Effector-Mediated PCD Suppression

The results indicate distinct patterns of PCD suppression by various effectors. Effectors with consistent anti-PCD activity, such as Avr3F (689), RxLR (1540), and RxLR (1546), exhibited significant suppression of BAX-induced cell death across both indirect and direct assays in oil palm genotypes, including the susceptible genotype 48 and the less susceptible genotype 82. These results align with previous studies, demonstrating that *Phytophthora* pathogens manipulate host innate immune responses by secreting multiple RxLR effectors, ultimately facilitating pathogen colonization [53].

Additionally, phylogenetic analysis and motif predictions revealed distinct clades among the effectors, suggesting variations in their functional characteristics. For example, Avr3F (689) and RxLR (1540) from the first major clade exhibited robust anti-PCD activity in both oil palm genotypes. Similarly, RxLR (1546) from the second major clade showed significant anti-PCD activity, particularly in specific genotypes. The presence of conserved motifs, such as RXLR-dEER, and K and Y motifs in Avr3a (689), enhances the modulation of PCD. The W, Y, and L motifs are among the most conserved C-terminal motifs across the RXLR-dEER superfamily [30,54,55]. These conserved motifs in Avr3F (689), RxLR (1540), and RxLR (1546) underscore their critical role in regulating PCD in both susceptible and less susceptible oil palm materials. These findings significantly contribute to our understanding of effector-mediated PCD suppression, where these effectors can disrupt R protein-mediated interactions and inhibit cell death in oil palms.

### 4.2. Variable Anti-PCD Activity

Our results also revealed effectors with variable anti-PCD activity and differences in their interaction with various oil palm genotypes. For instance, REX1 (1340) exhibited significant anti-PCD activity in the susceptible genotype 48, but this effect was not as prominent in the less susceptible genotype 82. These findings suggest that REX1 may be more effective in suppressing PCD in susceptible genotypes. This observation aligns with the work of Evangelisti et al. [27], who demonstrated that REX3, another *P. palmivora* effector, effectively interferes with host secretion processes, promoting root infection. The inhibition of host secretion by REX effectors is a common strategy shared by bacterial and oomycete pathogens.

REX3 is a conserved *P. palmivora* effector that targets the secretory pathway and enhances susceptibility, similar to REX2 and REX3. Isolate-specific variants of REX1 and REX4 may offer advantages in colonizing hosts other than *Nicotiana benthamiana* [27]. These insights expand our understanding of effector-mediated PCD suppression and the variations in resistance among oil palm genotypes.

Furthermore, our phylogenetic analysis identified a close relationship between REX1 (1340) and RxLR (1546) within the second major clade, with both showing significant anti-PCD activity in the susceptible genotype 48. However, this activity was less pronounced in the less susceptible genotype 82, suggesting that the role of REX1 in suppressing PCD may depend on the genetic background of the oil palm genotype. The distinctive RXLR-dEER motif likely contributes to this functionality, as noted in prior research [30]. The absence of the conserved W motif in their C-terminal domains may explain why these effectors suppress PCD in susceptible materials but are less effective in less susceptible materials, as observed with Avr1b [48].

Additionally, research on Avr3a [56] has shown that the C-terminal domain of *Phytophthora infestans* effectors is crucial for interacting with host proteins, such as CMPG1, and suppressing PCD. Our findings are consistent with these insights and further elucidate the role of effectors in PCD regulation, particularly in their interactions with different oil palm genotypes.

### 4.3. Unique Effector Behavior

Our study reveals distinctive behavior patterns among oil palm effectors. This category is characterized by limited anti-PCD activity, exemplified by effectors such as RXLR (1350, 1548, and 1550). Another category includes effectors that exhibit no anti-PCD activity, among which we find RxLR (896, 1033, and 1552).

Specific effectors, such as RxLR (1550) and Avr (1350), demonstrated anti-PCD activity in the susceptible material 48 but not in the less susceptible material 82, reflecting the variable roles effectors can play across genotypes. This divergence is likely influenced by specific motifs, such as the RXLR-dEER motif, which was particularly notable (Figure 3B). In contrast, RxLR (896) and RxLR (1033), which lack anti-PCD activity, primarily contain the RxLR motif. These findings highlight the diverse roles of RxLR effectors and suggest that their ability to regulate PCD may depend on the genetic background of the oil palm genotype [57,58].

Our results demonstrate that effector-induced PCD regulation varies between susceptible and less susceptible oil palm genotypes, likely due to genetic differences, including the presence or absence of specific R proteins. R proteins are critical components of plant defense, detecting pathogen effectors and initiating PCD to contain pathogen spread [26]. Understanding these effector–plant interactions is crucial for developing strategies to improve oil palm health and resilience. Identifying essential R proteins could lead to targeted breeding or genetic engineering approaches to enhance oil palm resistance by introducing or strengthening specific R proteins [59,60,61].

This study provides valuable insights into the functional diversity and specificity of effector-mediated PCD regulation across different oil palm genotypes. Understanding the interactions between pathogen effectors and host defense pathways is essential for developing strategies to enhance oil palm health and increase resistance to diseases such as bud rot. Future research could further investigate the molecular mechanisms behind the anti-PCD activities of these effectors and their interactions with host proteins. Additionally, exploring responses in a broader range of oil palm genotypes could provide a more comprehensive understanding of effector-mediated PCD regulation and support the development of targeted strategies for improving oil palm resistance to pathogens.

## Figures and Tables

**Table 1:** Indirect and direct essays for suppression of BAX-mediated programmed cell death (PCD) by different effectors in oil palm genotypes.

		Indirect Assay				Direct Assay		
Genotype *	Effector	(BAX + EV)/Ev ^(a)^	(Effector)/EV ^(a)^	Ratio ^(b)^	*p* Value ^(c1)^	(BAX + effector + EV)/(BAX + EV) ^(a)^	*p* Value ^(c2)^	Anti-PCD activity ^(d)^
48	689-690-Avr3F	0.82 ± 0.08	1.14 ± 0.88	1.39	*p* < 0.05	1.07 ± 0.41	*p* < 0.01	Yes
	896-897-RxLR	0.82 ± 0.08	0.80 ± 0.45	0.98	*p* > 0.01	0.71 ± 0.26	*p* < 0.01	No
	1033-1034-RxLR	0.82 ± 0.08	0.80 ± 0.38	0.97	*p* > 0.01	0.73 ± 0.14	*p* > 0.01	No
	1340-1341-REX1	0.82 ± 0.08	1.12 ± 0.42	1.36	*p* < 0.01	1.04 ± 0.38	*p* < 0.01	Yes
	1350-1351-Avr	0.82 ± 0.08	1.32 ± 0.42	1.61	*p* < 0.05	1.10 ± 0.27	*p* < 0.01	Yes
	1540-1541-RxLR	0.82 ± 0.08	1.38 ± 0.29	1.68	*p* < 0.01	1.06 ± 0.39	*p* < 0.05	Yes
	1546-147-RxLR	0.82 ± 0.08	1.25 ± 0.32	1.53	*p* < 0.01	1.15 ± 0.27	*p* > 0.01	Yes
	1548-1549-RxLR	0.82 ± 0.08	1.03 ± 0.40	1.25	*p* > 0.1	0.77 ± 0.37	*p* > 0.1	No
	1550-1551-RxLR	0.82 ± 0.08	1.44 ± 0.25	1.76	*p* < 0.01	1.01 ± 0.41	*p* < 0.01	Yes
	1552-1553-RxLR	0.82 ± 0.08	0.81 ± 0.35	0.98	*p* < 0.05	0.79 ± 0.23	*p* < 0.01	No
82	689-690-Avr3F	0.73 ± 0.22	1.06 ± 0.51	1.45	*p* < 0.01	1.20 ± 0.45	*p* < 0.01	Yes
	896-897-RxLR	0.73 ± 0.22	0.70 ± 0.15	0.96	*p* < 0.01	0.63 ± 0.20	*p* < 0.01	No
	1033-1034-RxLR	0.73 ± 0.22	0.70 ± 0.18	0.96	*p* < 0.01	0.63 ± 0.28	*p* < 0.01	No
	1340-1341-REX1	0.73 ± 0.22	0.78 ± 0.29	1.07	*p* < 0.05	0.62 ± 0.39	*p* < 0.01	No
	1350-1351-Avr	0.73 ± 0.22	0.69 ± 0.22	0.95	*p* < 0.1	0.59 ± 0.30	*p* < 0.01	No
	1540-1541-RxLR	0.73 ± 0.22	1.28 ± 0.42	1.76	*p* < 0.05	1.01 ± 0.25	*p* < 0.01	Yes
	1546-147-RxLR	0.73 ± 0.22	1.14 ± 0.20	1.57	*p* < 0.05	1.10 ± 0.32	*p* < 0.01	Yes
	1548-1549-RxLR	0.73 ± 0.22	0.69 ± 0.20	0.95	*p* < 0.01	0.63 ± 0.23	*p* < 0.01	No
	1550-1551-RxLR	0.73 ± 0.22	0.71 ± 0.30	0.98	*p* > 0.1	0.70 ± 0.17	*p* < 0.01	No
	1552-1553-RxLR	0.73 ± 0.22	0.71 ± 0.18	0.98	*p* > 0.1	0.68 ± 0.27	*p* > 0.1	No

^(a)^ Average ratio and standard error calculated from the number of blue spots counted from 15 co-bombarded leaflet segments. ^(b)^ Ratio from indirect assay is calculated as (Effector/Ev ratio)/(BAX/Ev ratio). EV: empty vector. ^(c1)^ *p* value for indirect assay calculated using Wilcoxon rank sum test. ^(c2)^ *p* value for direct assay calculated using Wilcoxon signed-rank test. ^(d)^ The anti-PCD activity of each gene is determined by whether the ratios for direct and indirect assays are statistically significantly greater than 1.0. * Genotype 48 is a cultivar of *E. guineensis* D × D Code 48, which is known to be susceptible in the field, and genotype 82 is a cultivar of *E. guineensis* D × D Code 82, which is considered less susceptible (possibly resistant) in the field.

## Data Availability

The original contributions presented in the study are included in the article/Supplementary Material. Further inquiries can be directed to the corresponding author.

## Supplementary Materials

The following supporting information can be downloaded at: https://www.mdpi.com/article/10.3390/jof10110750/s1, Figure S1: suppression of BAX-mediated PCD by diverse predicted effectors: suppression or induction of BAX-triggered cell death by 10 effectors in two genotypes of oil palm: susceptible (48) and less susceptible (82); Table S1: cytoplasmatic effector summary information and primer information.
