# Supplementary figures and images for: Effector-Mediated Suppression of Programmed Cell Death by Phytophthora palmivora in Oil Palm
Source: J Fungi (Basel). 2024 Oct 30;10(11):750. doi: 10.3390/jof10110750 (PMC11595804; doi:10.3390/jof10110750)

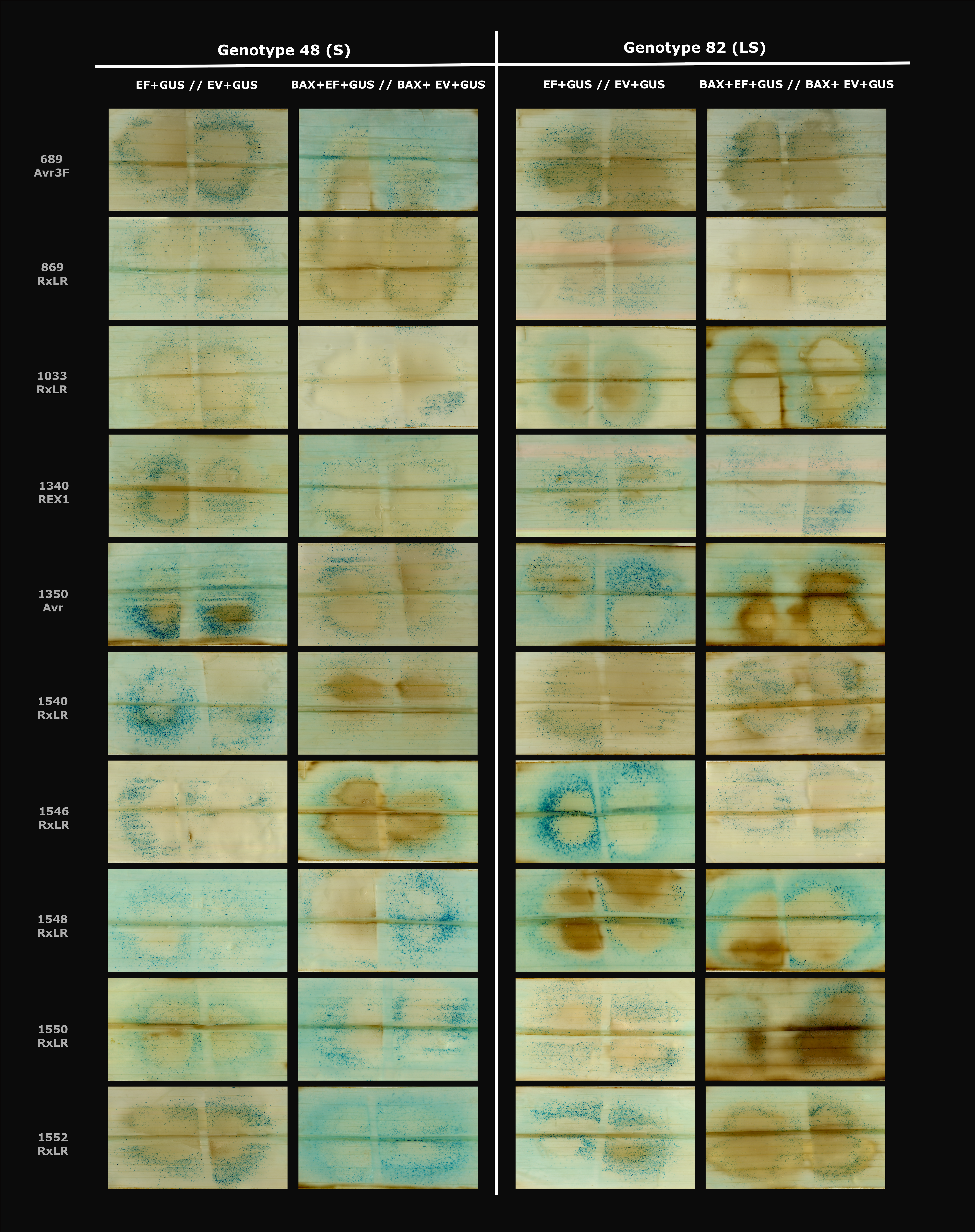

Supplement: Supplementary file 1 [file jof-10-00750-s001.zip › Suppplementary/Figure S1.tiff]
